# Decoding airway granulogenesis in children: unveiling risk factors for tracheobronchial foreign body aspiration and complications

**DOI:** 10.1186/s13052-025-01869-0

**Published:** 2025-01-27

**Authors:** Yuting You, Meili Shen, Li’e Zeng, Jingyang Zheng

**Affiliations:** 1 Children’s Respiratory Department, Quanzhou Maternity and Children’s Hospital, Quanzhou, Fujian 362000 China; 2 Children’s Critical Care Medicine Department, Quanzhou Maternity and Children’s Hospital, Quanzhou, Fujian 362000 China

**Keywords:** Exogenous foreign body, Bronchoscopy, Granulomatous tissue, Pediatrics

## Abstract

**Background:**

Exogenous foreign body aspiration is a common high-risk condition in children. In a few cases, foreign body aspiration can lead to airway granulomas that interfere with tracheoscopic foreign body removal and threaten the life of the child.

**Methods:**

This study was a retrospective analysis of the clinical data of 184 pediatric patients who were admitted to Quanzhou Children’s Hospital from 2018 to 2021 with exogenous tracheobronchial foreign bodies.

**Results:**

Respiratory foreign bodies tend to occur during the winter and spring seasons. The solid foreign bodies were mostly nut foreign bodies, the location of implantation was the left lung rather than the right lung in many patients, and complications such as pulmonary atelectasis, emphysema, mediastinal and subcutaneous emphysema, and granulomatous tissue formation were noted in these patients. Statistical models suggested that the time of foreign body impaction and the release of oil were risk factors for tracheal granulation, with the logistic model presenting an AUC of 0.948, precision of 0.676, and sensitivity of 0.895, whereas the XGBoost model presented an AUC of 0.902, precision of 0.912, and sensitivity of 0.875.

**Conclusions:**

Tracheobronchial foreign bodies primarily develop in male children under the age of 3 and often lead to various complications. The time of foreign body insertion and the release of oil from the obstructed foreign body have been identified as high-risk factors for the development of tracheobronchial granulation tissue. When the time of foreign body insertion without oil release exceeds 99.98 h or when the time of foreign body insertion with oil release exceeds 47.94 h, tracheobronchial granulation formation strongly suggests that the child is at high risk of developing airway granulation. In such cases, family members must implement increased supervision of the child to prevent choking. Medical professionals should obtain a detailed medical history of the affected child and accordingly select the most appropriate method to promptly remove the foreign body to resolve the issue of airway obstruction and reduce the likelihood of pulmonary complications in the child.

## Background

Airway foreign bodies are common but easily overlooked health problems in children and are important causes of respiratory distress and choking in children [1]. These foreign bodies can be broadly classified into two categories: exogenous (from external sources) and endogenous (produced in the body) [2]. Whereas there are a wide variety of exogenous solid airway foreign bodies, each material has unique physical and chemical properties that may lead to varying degrees of injury [3]. The complications of solid airway foreign bodies are diverse and include pneumonia, emphysema, pulmonary atelectasis, pneumothorax, mediastinal emphysema and tracheal granulation tissue formation [3–5].

The retention of foreign bodies in the airways can damage the airway mucosa, and local fibroblast and vascular endothelial cell proliferation can lead to granulation tissue formation [6], which is a particularly worrying outcome because it may lead to poor detection of foreign bodies via tracheoscopy, resulting in missed diagnoses and long-term airway damage. However, the exact mechanism by which exogenous solid airway foreign bodies induce granulation tissue formation is not fully understood. Some researchers [7–9] have suggested that it may be related to the size, shape, material composition and duration of retention of the foreign body.

Our clinical work revealed that certain characteristics of exogenous solid foreign bodies, such as the ability to release oils, may predispose the airway to granulation tissue formation; i.e., oils may act as potent irritants, exacerbating inflammatory responses and promoting tissue proliferation. In addition, we observed that the foreign body retention time is a key factor leading to airway mucosal injury and edema, which is in agreement with the findings of the study by Lin FZ et al. [10]. The longer the foreign body retention time was in our study, the greater the risk of granulation formation.

To validate the findings of these clinical papers, we retrospectively analyzed clinical data from 184 children diagnosed with exogenous solid tracheobronchial foreign bodies to elucidate factors associated with airway granuloma formation. Our primary aim was to determine the optimal method of tracheoscopic foreign body removal to improve removal rates, minimize complications, and reduce lung injury. We hope to contribute to the development of more effective clinical strategies for the management of airway foreign bodies in children.

## Materialsand methods

### Clinical data

This study involved a retrospective analysis of the clinical data of 184 children who were diagnosed with exogenous tracheobronchial foreign bodies and admitted to Quanzhou Children’s Hospital between January 2018 and December 2021. The analyzed data included information on on sex, age, type of foreign body, location of the foreign body embedded in the trachea, pulmonary complications, and intratracheal granulomatous proliferation in the children.

### Diagnostic criteria

Solid foreign bodies were diagnosed using tracheoscopy.

### Methods

After communication regarding the patient’s condition, the family of each patient signed the informed consent form for participation in the study. Routine electrocardiogram, blood routine, coagulation function, and tests for detecting HIV, syphilis antibodies, hepatitis B virus, hepatitis C virus, and other blood-borne disease-related pathogens wereperformed. Each patient was instructed to fast prior to the operation and abstain from food and water for a period of 6–8 h. In addition, 5% dextrose solution was administered intravenously to each patient [2]. The appropriate outer diameter of the bronchoscope was selected according to the age and weight of the child. The bronchoscope was subsequently inserted into the nasal cavity. Afterward, 1 mL of 2% lidocaine was administered for surface anesthesia of the pharynx, and after it passed through the vocal folds, another 1 mL of 2% lidocaine was administered for surface anesthesia of the endotracheal tubes using the “anesthesia and entry” technique. According to the condition of the trachea and the type of foreign body, once the foreign body was visualized, the appropriate foreign body basket or foreign body clamp was selected based on the characteristics of the foreign body and the size of any granulation tissue present. After the foreign body was removed, all levels of the trachea and bronchus were re-examined. Alveolar lavage was performed based on the secretion status, and the corresponding samples were collected for examination. The child’s respiration, heart rate, and transcutaneous oxygen saturation were monitored continuously throughout the procedure. The oxygen flow was adjusted according to the transcutaneous oxygen saturation to maintain a minimum oxygen saturation level of 0.92. After the operation, the child’s respiration, heart rate, and transcutaneous oxygen saturation were monitored continuously for another 3 h. Patients were then administered oxygen therapy, with an appropriate oxygen flow rate selected according to their oxygen saturation levels and adjusted as required during the therapy.

### Statistical analysis

R statistical software 4.1.3 was employed for modeling by setting up the code. The integrated development environment tool R Studio (version number 2022.02.1 Build 461) was employed for data modeling analysis and output. Two models, namely, the logistic regression model and the extreme gradient boosting (XGBoost) model, were used for outcome prediction. Numerical data are expressed as proportions (%). ORs and 95% CIs were calculated using the logistic regression model, and the results were expressed as ‘significance’ using the XGBoost model, with larger values indicating a greater impact on the outcome. Receiver operating characteristic (ROC) curve analyses were performed using the factors associated with the two models that influenced the outcome, with an area under the curve (AUC) > 0.5 indicating a predictive value. Differences were considered statistically significant at a two-sided *P* < 0.05.

## Results

### Demographic characteristics

A total of 184 patients with exogenous tracheobronchial foreign bodies were included in the present study. Among these, 128 (69.57%) were males and 56 (30.43%) were females, resulting in a male-to-female ratio of 2.29:1. The age of these patients ranged from 10 months to 10 years, with a median age of 19 months.

### Foreign body type

**Fig.1:**
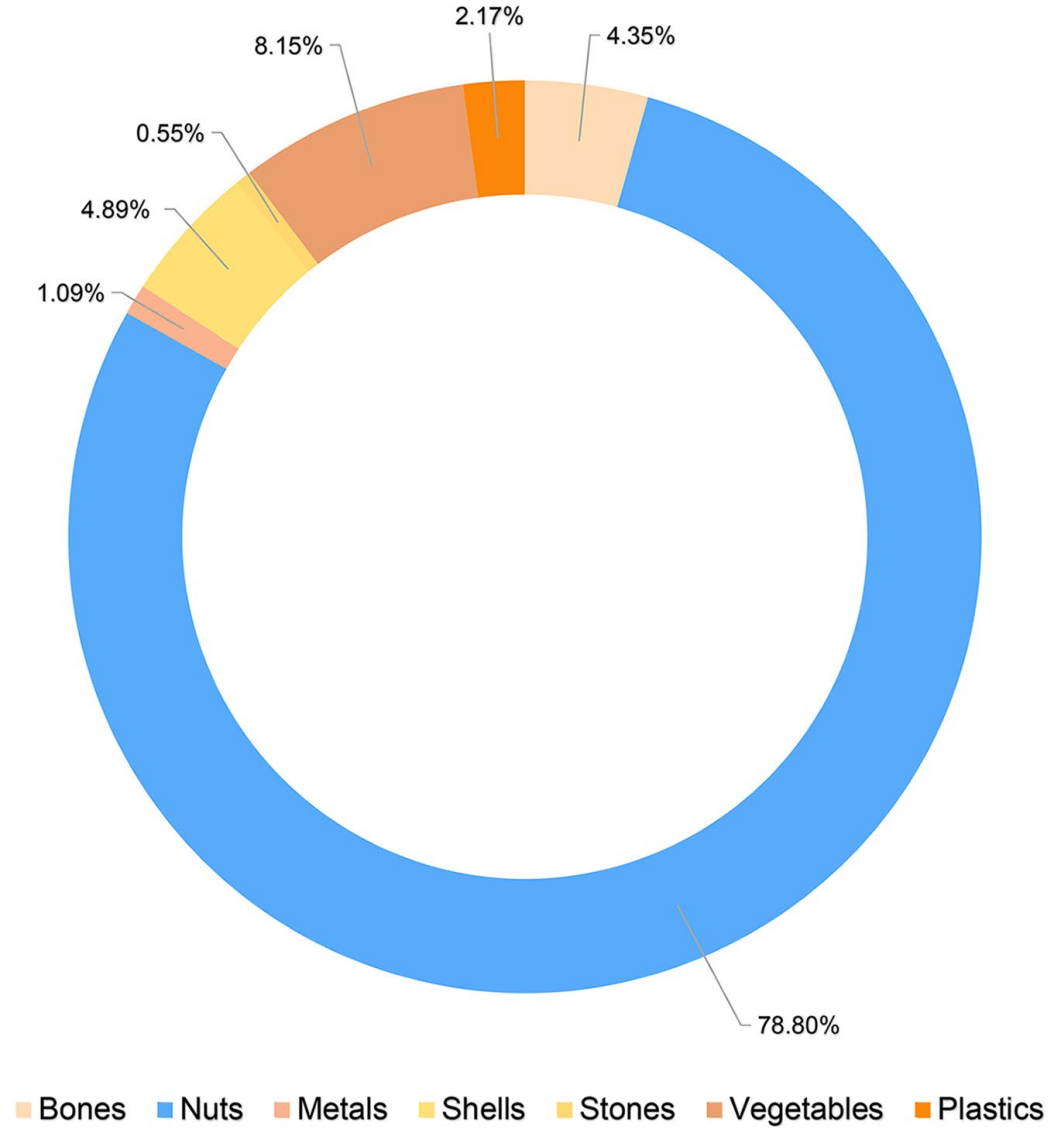
Solid foreign body type

The most common form of exogenous foreign body among the included cases was nut-like foreign bodies (releasing of oil and grease foreign bodies), which accounted for 145 cases (78.8% of the cases). The next most common exogenous foreign bodies were fruits and vegetables, various types of shells, and bones, which accounted for 8.15%, 4.89%, and 4.35% of all cases, respectively. Plastic, metal, and stone foreign bodies were less frequently observed, accounting for 2.17%, 1.09%, and 0.55% of all cases, respectively (Fig. 1).

### Distribution of exogenous solid impact sites and seasons

**Fig. 2:**
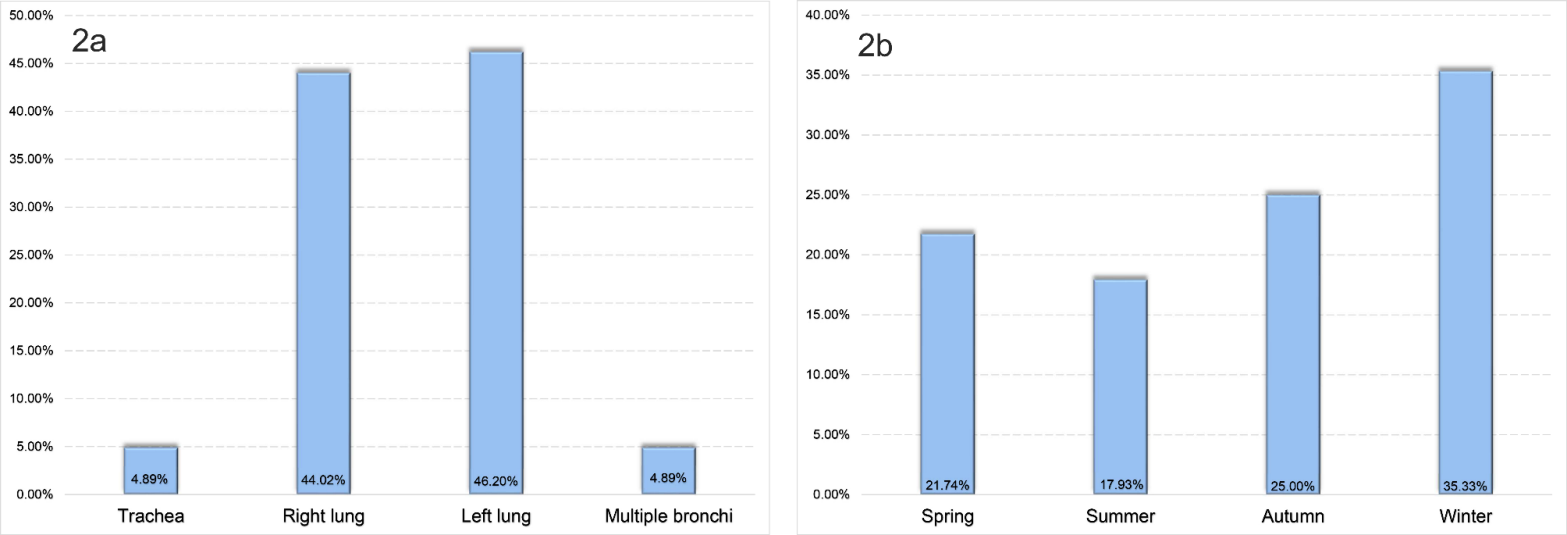
**a**: Location of foreign body; **b**: Season

The 184 cases analyzed presented the following distribution of the primary locations where the foreign bodies had lodged in the airway: the main trachea in 9 cases (4.89%), the right lung in 81 cases (44.02%), the left lung in 85 cases (46.20%), and multiple bronchi in 9 cases (4.89%). Among the patients whose bronchi were the primary location, those whose left bronchus was affected were slightly more common than those whose right bronchus was affected. In addition, the left and right main bronchi were the most frequently involved sites (Fig. 2a). The occurrence of these incidents demonstrated a seasonal pattern, with a higher frequency observed during the winter and spring seasons than during the summer and autumn months. Specifically, 40 cases (21.74%) occurred in spring (March-May), 33 cases (17.93%) occurred in summer (June-August), 46 cases (25.00%) occurred in autumn (September-November), and 65 cases (35.33%) occurred in winter (December-February) (Fig. 2b).

### Exogenous solid foreign body complications

**Fig. 3:**
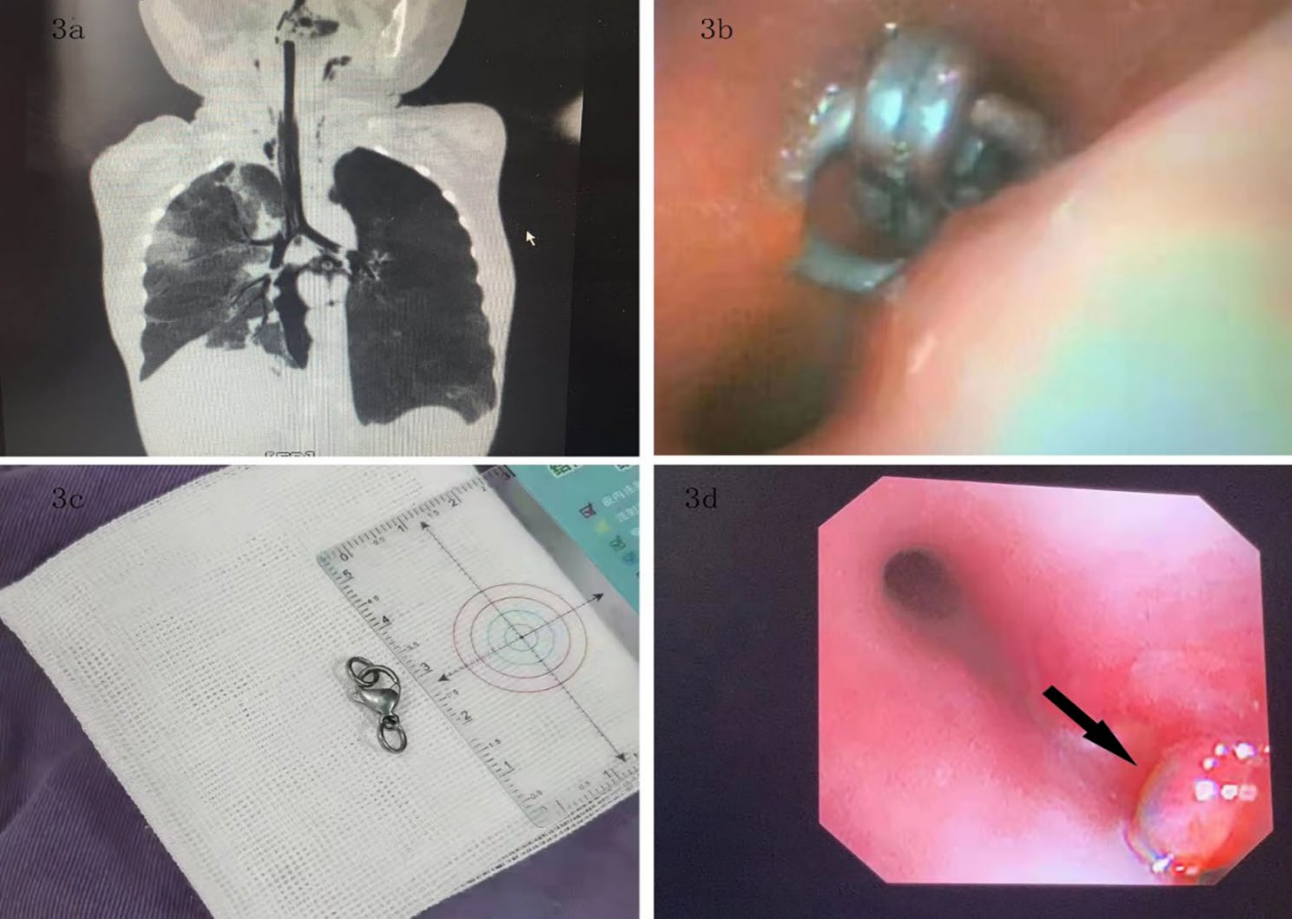
**a**: Emphysema and mediastinal emphysema on lung CT after choking on a solid foreign body; **b**: The tracheoscopic view of a metallic foreign body in the opening of the right main bronchus; **c**: The foreign object is a metal necklace clasp; **d**: Granulomatous hyperplasia of the right main bronchial opening visible after the removal of the foreign body

In the present study, the following complications related to exogenous solid foreign bodies were revealed in lung CT and bronchoscopy: emphysema in 65 patients (35.33%), pulmonary atelectasis in 40 patients (0.09%), mediastinal emphysema/subcutaneous emphysema in 8 patients (4.35%), and granulomatous tissue hyperplasia in 144 patients (78.69%) (Table 1; Fig. 3a and d).

**Table 1:** Exogenous solid foreign body complications

Imaging and Bronchoscopy	Cases		(%)
Emphysema		65	35.33
Atelectasis		40	21.74
Subcutaneous emphysema /mediastinal emphysema		8	4.35
granulation tissue		144	78.69

### Factors influencing airway granulogenesis via solid particles

**Fig. 4:**
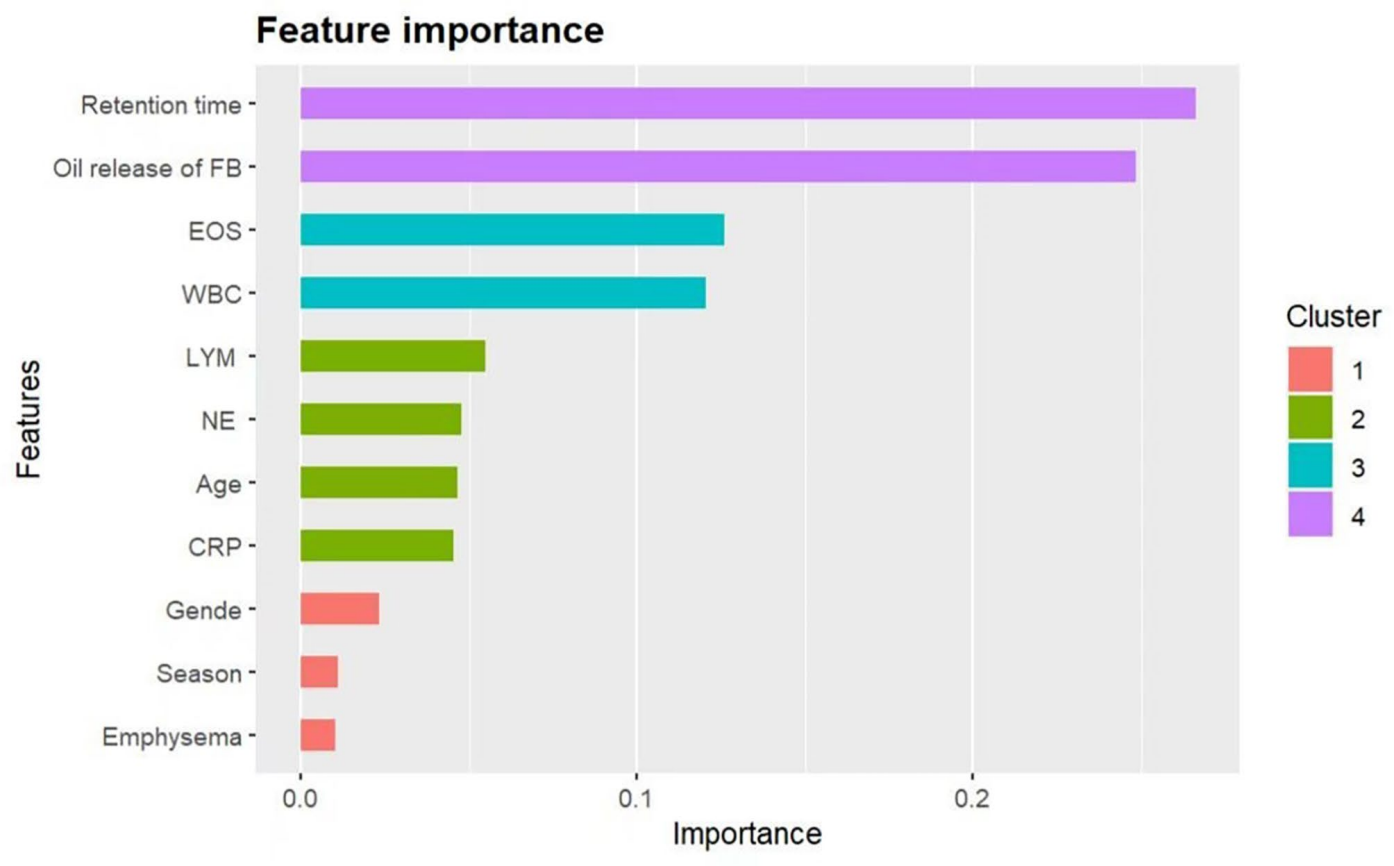
R-Language and XGBoost model variable importance rank chart. EOS eosinophil count; WBC white blood cell count; LYM lymphocyte count; NE neutrophil count

**Fig. 5:**
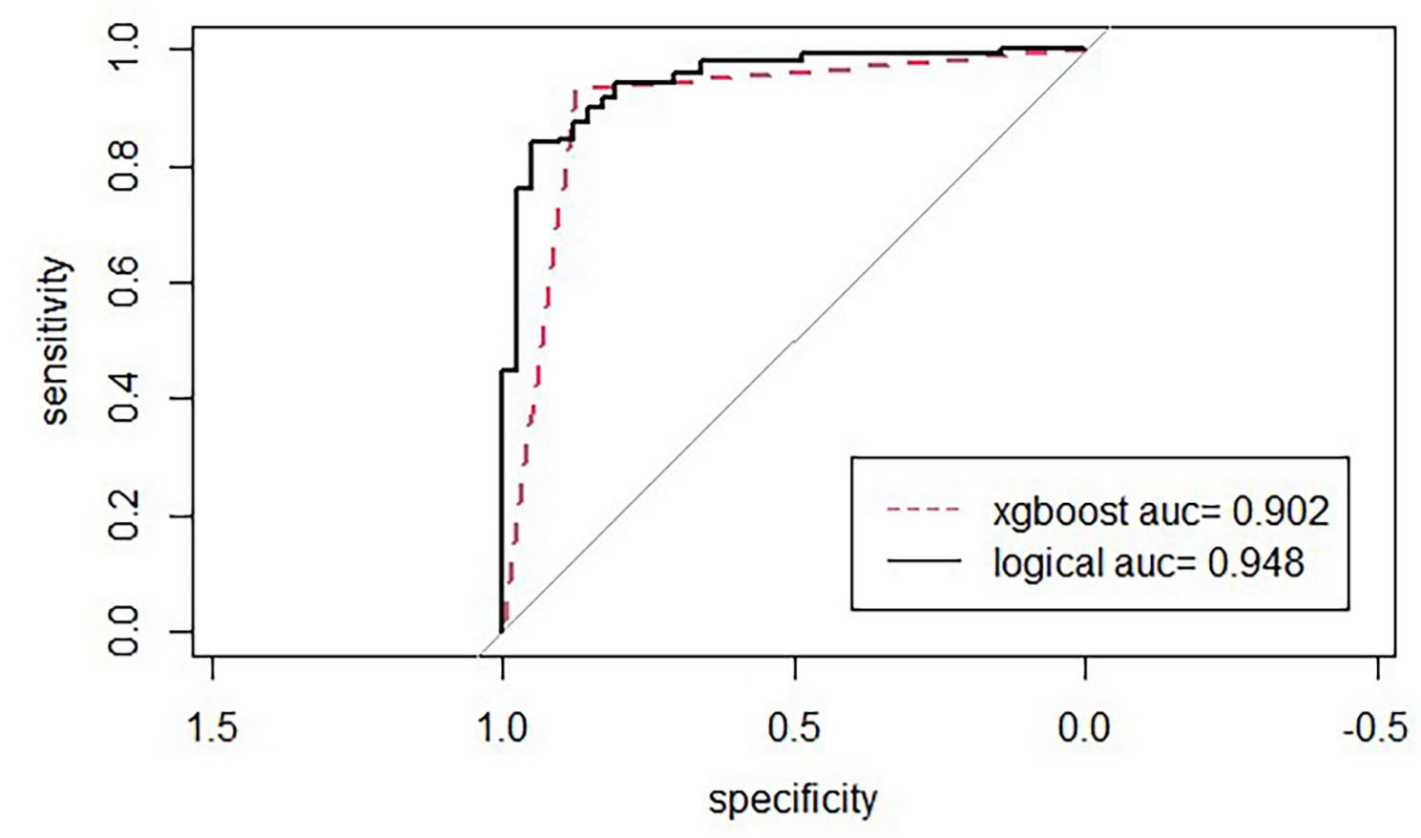
Comparison of the AUCs of the two models in R language

The R language logistic model and the XGBoost model were applied to model the factors affecting the formation of airway meatus. Several factors affect the formation of meatus. The logistic model suggested that the time of foreign body impact (OR = 1.984, 95% CI 1.543–2.631; *P* < 0.01) and whether the foreign body released grease (OR = 10.67, 95% CI 4.753–24.96; *P* < 0.01) were the main risk factors (Table 2). The XGBoost model suggested that the time to foreign body impaction (significance = 0.266), whether the foreign body released oil (significance = 0.249), and the absolute value of eosinophils (significance = 0.126) were the three most important variables (Fig. 4). According to the logistic model, the AUC was 0.948, the precision was 0.676, and sensitivity was 0.895. According to the XGBoost model, the AUC was 0.902, the precision was 0.912, and the sensitivity was 0.875 (Fig. 5). A time threshold was derived as a predictor of combined airway granulomas. According to this time threshold, when the time of foreign body impaction with oil release was ≥ 99.98 h and the time of foreign body impaction without oil release was ≥ 47.94 h, it suggested the possibility of combined airway granulomas.

**Table 2:** Multivariate R logistic regression analysis of respiratory granulogenesis

Variable	Z	Bate	OR	95%CI	*P*
Gender Age	0.585 0.137	0.221 0.021	1.247 1.021	(0.584,2.584) (0.776,1.443)	0.558 0.891
Retention time	5.063	0.685	1.984	(1.543,2.631)	0.000
Oil release of FB	5.623	2.367	10.67	(4.753,24.96)	0.000

## Discussion

Children encounter a diverse set of foreign objects, and the specific location in which these objects become lodged in their bodies is subject to various factors. In line with previous investigations conducted by Na’ara S et al. [11], the present study revealed a greater occurrence of tracheobronchial foreign bodies among children younger than 3 years, with males being more affected than females at a ratio of 2.29:1. These findings are consistent with those reported in most previous studies [12–14]. The difference noted between the two genders could have stemmed from the heightened inquisitiveness and physical engagement of male children with their surrounding environment [15]. Solid foreign bodies accounted for 78.97% of all cases and were the predominant type of airway foreign body incident. Nuts, particularly peanuts and melon seeds, accounted for the greatest number of cases. These incidents were more prevalent during the winter and spring seasons. This pattern could be related to the fact that these months represent a period of traditional festivities in China when children have greater access to nuts such as peanuts and melon seeds [16]. These incidents are often a result of inadequate supervision by family members or improper feeding practices adopted by relatives. Moreover, the limited chewing ability of young children, coupled with activities such as running, laughing, and crying during meals, also contributes to the risk of airway foreign bodies. Therefore, family members of young children must exercise heightened vigilance to monitor whether their children are accessing or consuming food items that may cause airway obstruction. It is also imperative to enhance supervision of children accessing other potential objects that may become lodged in the airway.

Interindividual anatomical variations may lead to significant differences in the morphology of the left and right main bronchi. The left main bronchus has a characteristic slender structure, inclined to the longitudinal axis of the trachea at an angle of about 37.5 degrees. The right main bronchus, on the other hand, is thick, short, and steep, with an angle of about 23 degrees from the longitudinal axis of the trachea. The right main bronchus appears notably thicker, shorter, and straighter than its left counterpart, with the tracheal carina exhibiting leftward displacement [17]. These anatomical differences contribute to the increased vulnerability of the right main bronchus to the aspiration of foreign objects. Interestingly, the findings of the present study aligned with those reported previously by Ahmed and Shuiabu [18], who reported a slightly greater incidence of foreign body aspiration on the left side (46.20%) than on the right side (44.02%). Nevertheless, the conflicting findings reported in the literature suggest that the prevalence of impacted foreign bodies in the bronchi is either similar between the left and right sides or slightly more frequent on the left side in the Chinese population [19, 20]. The following could be the reasons for this:

1. Children exhibit immature bronchial development, and the difference in the inclination of the left and right main bronchi between children and adults is not as evident. At a certain age, this inclination might even become equal on both sides. The type, shape, and size of a foreign body also influence its entry into the child’s body. A foreign body with a large volume tends to lodge in the trachea, whereas a relatively smaller body is more likely to enter the main bronchus or deeper parts on both sides.
2. The position of the child while he/she is choking on the foreign body is another important factor to consider. The probability of the foreign body entering the right or left bronchus is different depending on whether the child is in an upright or oblique lateral position while choking. The right main bronchus appears notably thicker and shorter, so the foreign body can easily enter the right side in the upright position. In the oblique position, depending on the current position, it is not possible to predict which side of the bronchus a foreign body will tend to enter.
3. The initial position of the foreign body within the tracheobronchial tube varies with time due to coughing, crying, or changes in the body position of the child, which may cause the foreign body to become dislocated [21].

Pneumonia, obstructive emphysema, pulmonary atelectasis, mediastinal emphysema, subcutaneous emphysema, granulomatous hyperplasia, etc., are the major complications of exogenous solid airway foreign bodies. Wang Y, et al. reported thin-slice CT as the best method for locating lodged foreign bodies, with an accuracy as high as 99.4% [22]. In the present study, a CT scan of the lungs of children revealed the following as the main comorbidities of foreign body aspiration: emphysema in 65 cases (35.33%), atelectasis in 40 cases (21.74%), subcutaneous emphysema/mediastinal emphysema in 8 cases (4.35%), and granulation tissue hyperplasia in 144 cases (78.69%). These complications caused by solid foreign bodies were not treated further, except for post-operative nebulized inhalation. The follow-up after a period of 2 weeks to 1 month revealed that all patients had been cured, except for 1 patients in which the granulation tissue was significantly enlarged, and symptoms of dyspnea were observed at the follow-up. The patient’s dyspnea was relieved after nebulized inhalation and intravenous glucocorticoid treatment. This observation indicated that treating airway obstruction could resolve the occurrence and development of complications. This would be important for prognosis. Therefore, bronchoscopy could be the most direct method for confirming the diagnosis and treatment of tracheobronchial foreign bodies [15, 23].

The bacterial culture examination was also performed for the 184 patients whose bronchoalveolar lavage fluid was used in the present study. The positive rate of bacterial culture was 15.76% (29/184). The most common bacteria isolated were *Acinetobacter baumannii*, which accounted for 27.57% (8/29) of the isolates, followed by *Streptococcus pneumoniae* [24.14% (7/29)], *Klebsiella pneumoniae* [20.69% (6/29)], *Enterobacter cloacae* [10.34% (3/29)], *Staphylococcus aureus* [6.90% (2/29)], *Haemophilus influenzae* [3.45% (1/29)], *Pseudomonas aeruginosa* [3.45% (1/29)], and *Burkholderia cepacia* complex [3.45% (1/29)]. The top three pathogens were *Acinetobacter baumannii*, *Streptococcus pneumoniae*, and *Klebsiella pneumoniae*, which is consistent with the reported distribution of common pathogens causing community-acquired pneumonia in children [24]. The results of the statistical analysis indicated the presence of a diverse set of infecting pathogens, including opportunistic pathogens. The author suggested that this could be related to the translocation and implantation of common respiratory tract parasites after foreign body aspiration or the presence of pathogenic bacteria carried by the foreign body itself. The early identification of infecting pathogens provides a basis for the rational use of antibiotics, which could reduce the risk of secondary infections resulting in pneumonia, lung abscess, lung necrosis, and other complications.

Exogenous solid foreign body insertion may result in the formation of airway granulation. The proliferation of granulation tissue may envelop the foreign body, making it difficult to identify the foreign body during bronchoscopy, which increases the abundance of granulation tissue, leading to misdiagnosis and leakage. The close relationship between the granulation tissue and the foreign body may cause local bleeding and mucosal swelling during the removal process. In patients with a significant amount of bleeding, rapid obstruction of the airway may occur, leading to asphyxiation and life-threatening situations. Therefore, the removal of the foreign body becomes further challenging in such cases [25].

Thomaskea reported the type of foreign object inhaled and the duration of its presence in the respiratory tract as the most significant factors leading to complications, including granulation tissue hyperplasia [26]. However, Zhang Yueming et al. reported that the factors contributing to granulation tissue formation are further complex and exhibit varying degrees of correlation with the nature of the foreign object and its lipid content. Therefore, further research is needed in this regard [27]. In this context, clinical data on whether children with foreign body aspiration exhibited lipid release were collected in the present study, followed by the application of logistic regression models in R language and XGBoost to model and analyze the factors influencing the formation of granulation tissue in the airway. Modeling analysis revealed that the duration of foreign body impaction and whether the foreign body released oil were high-risk factors for the formation of airway granulation tissue. The logistic regression model presented an AUC of 0.948, along with high precision, sensitivity, and F value. The XGBoost model presented an AUC of 0.902, along with good precision, sensitivity, and F value. According to this analysis, a critical time threshold was determined to predict whether airway granulation tissue would be formed. The threshold was as follows: when the duration of impaction of the foreign body that released oil was ≥ 99.98 h, and the duration of impaction of the foreign body that did not release oil was ≥ 47.94 h, it indicated the possible formation of airway granulation tissue.

### Limitations of the present study

The present study has certain limitations that must be acknowledged. First, the data collected in the study were limited to the Quanzhou region in Fujian, which must have limited the comprehensiveness of the analysis in terms of the various types of solid foreign bodies. In addition, this study focused solely on the impact of oil release and the residence time of foreign bodies in the airway on the development of granulation tissue. However, other factors, such as the size, shape, and sharpness of the foreign bodies, could also play a significant role. Therefore, further refinement of these factors in future studies is necessary.

## Conclusion

Tracheobronchial foreign bodies are pediatric emergencies that typically occur in boys under the age of 3. These foreign bodies usually involve nuts, foreign objects that release oil and grease for more than 2 days, or foreign objects that do not release oil and grease for more than 4 days. Such incidents may result in the development of granulation tissue in the airways. It is, therefore, crucial that family members exhibit enhanced supervision of the child to prevent them from choking on foreign objects. In the event that a child chokes on a foreign body, seeking prompt medical treatment is essential. Medical professionals should be proactive in gathering a detailed history of the affected child to address the child’s airway obstruction immediately.

## Data Availability

The datasets used and analyzed during the current study can be available from the corresponding author on reasonable request.

## Abbreviations

AUC: Under the curve
WBC: White blood cell count
LYM: Lymphocyte count
NE: Neutrophil count
ROC: Receiver operating characteristic
